# Supplementary figures and images for: Conflicting phylogenetic signals in plastomes of the tribe Laureae (Lauraceae)
Source: PeerJ. 2020 Oct 15;8:e10155. doi: 10.7717/peerj.10155 (PMC7568859; doi:10.7717/peerj.10155)

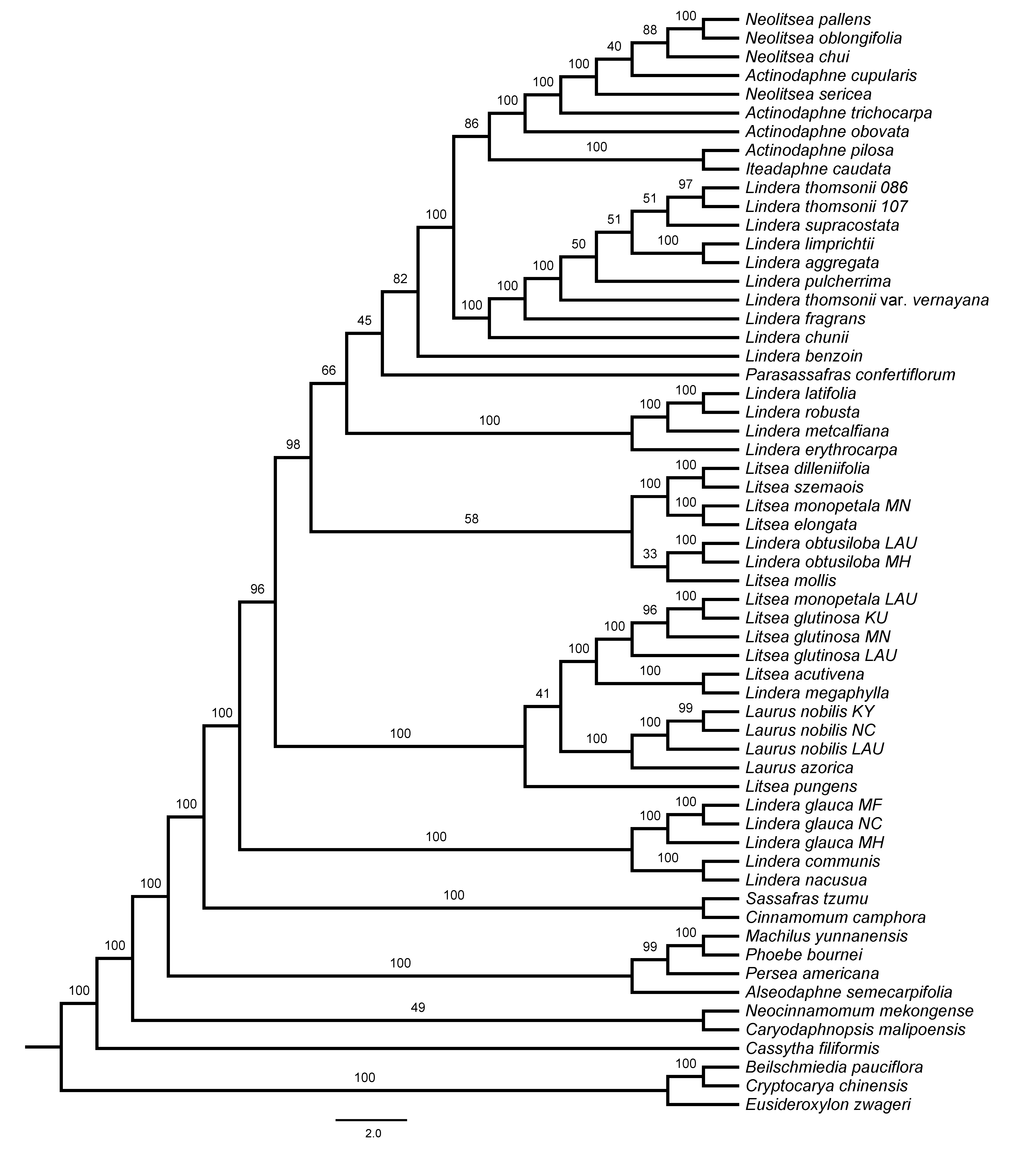

Supplement: Figure S1 — Bootstrap support is indicated on the branches. [file peerj-08-10155-s007.png]

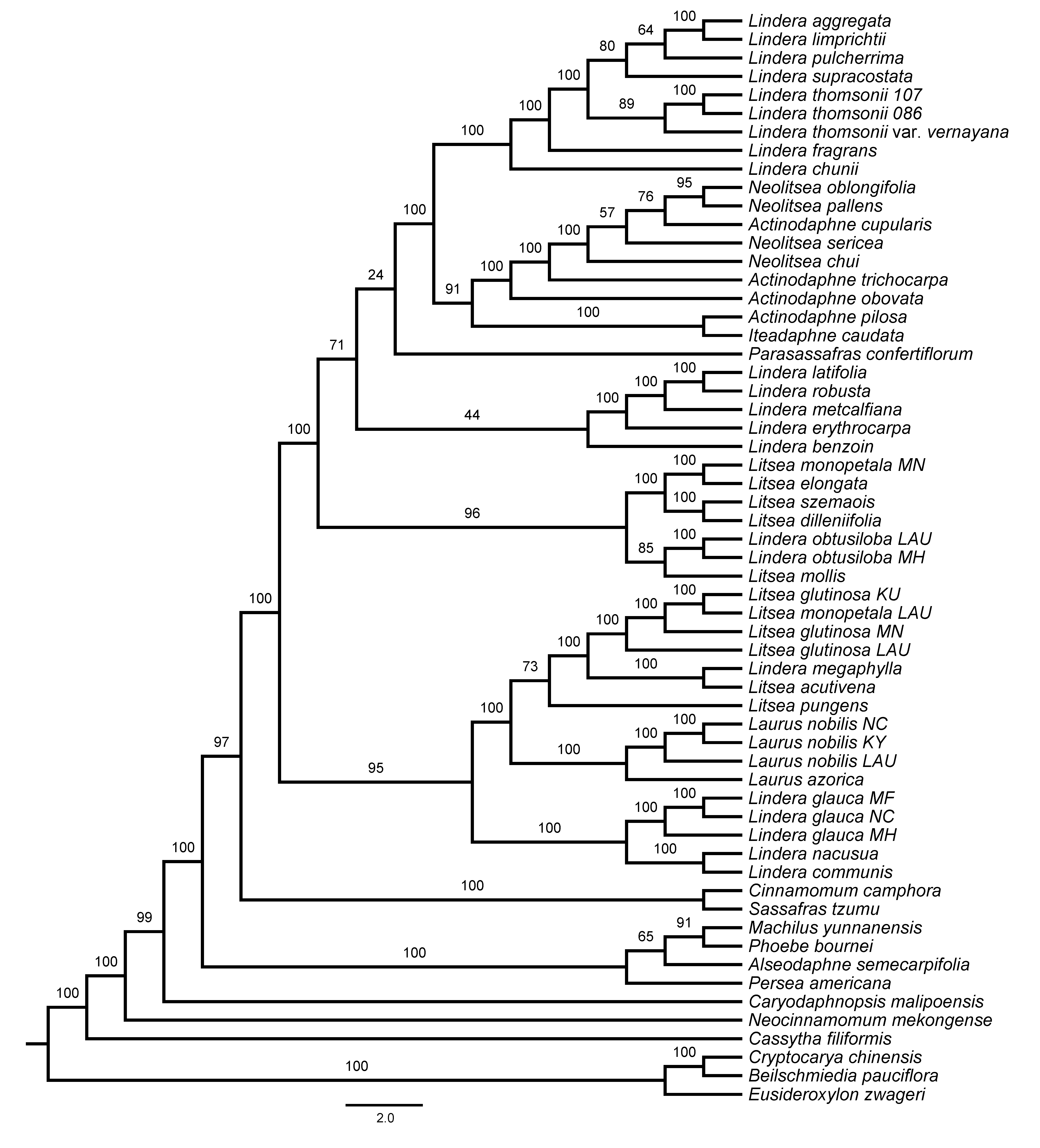

Supplement: Figure S2 — Bootstrap support is indicated on the branches. [file peerj-08-10155-s008.png]

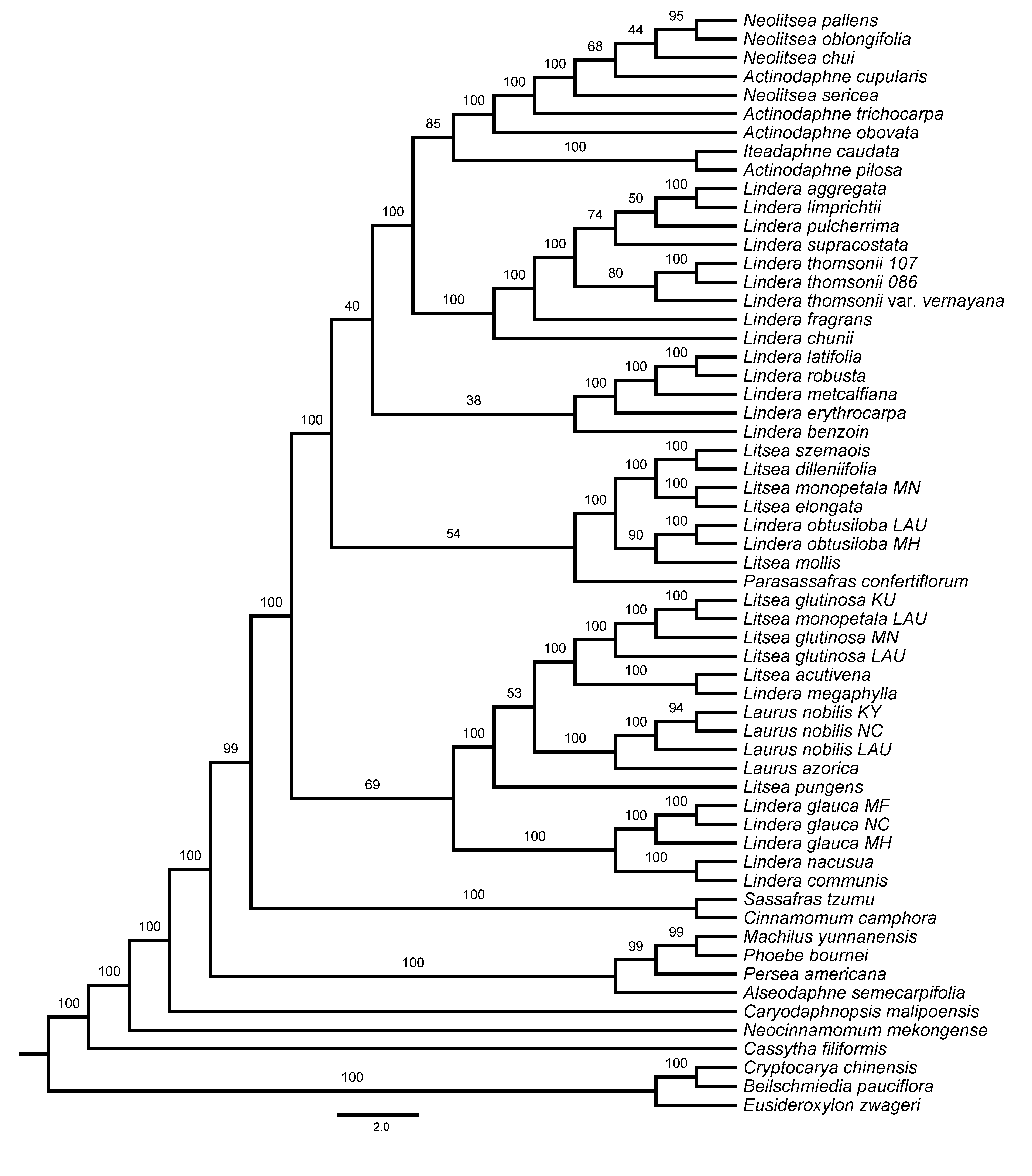

Supplement: Figure S3 — Bootstrap support is indicated on the branches. [file peerj-08-10155-s009.png]

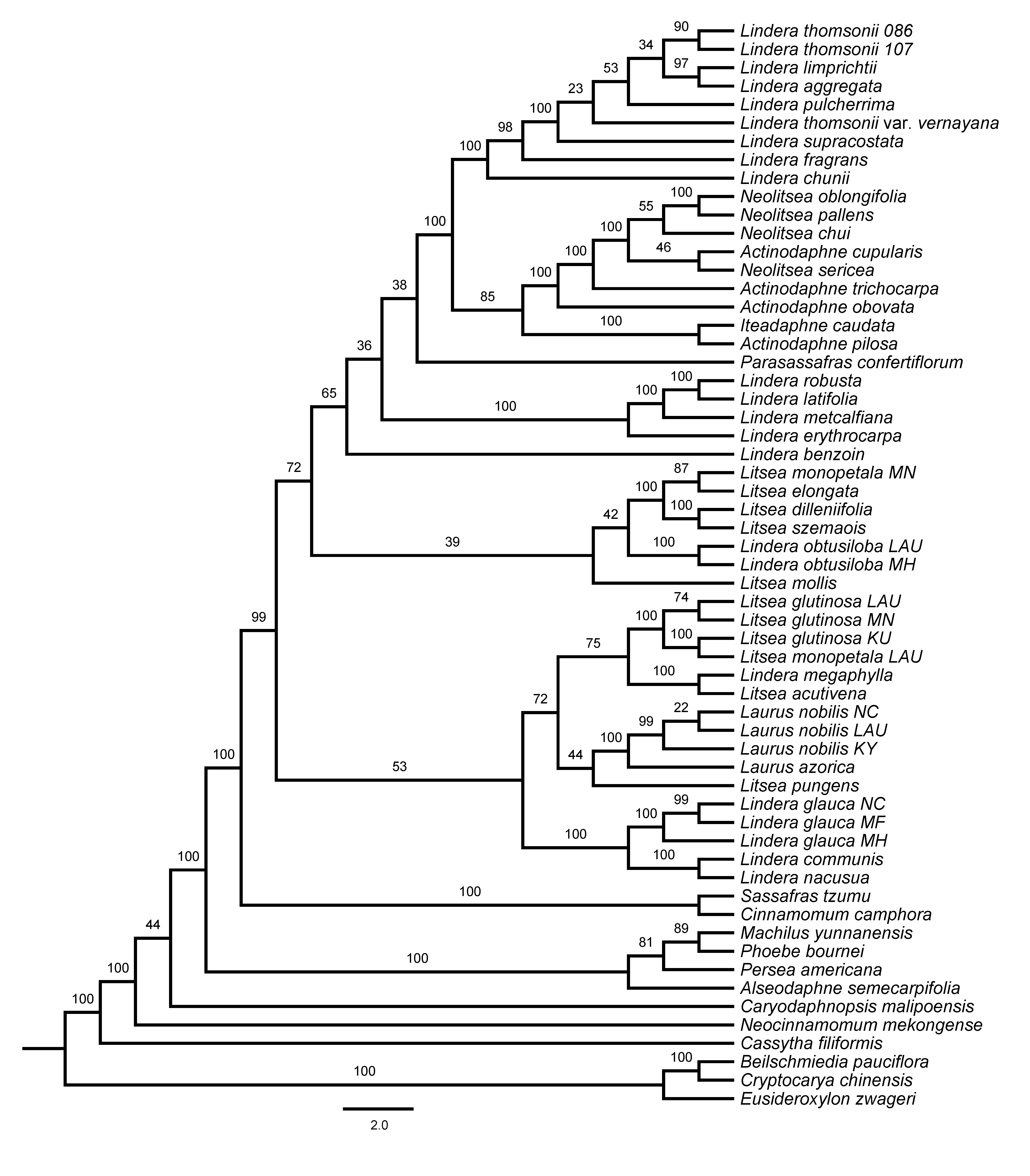

Supplement: Figure S4 — Bootstrap support is indicated on the branches. [file peerj-08-10155-s010.png]

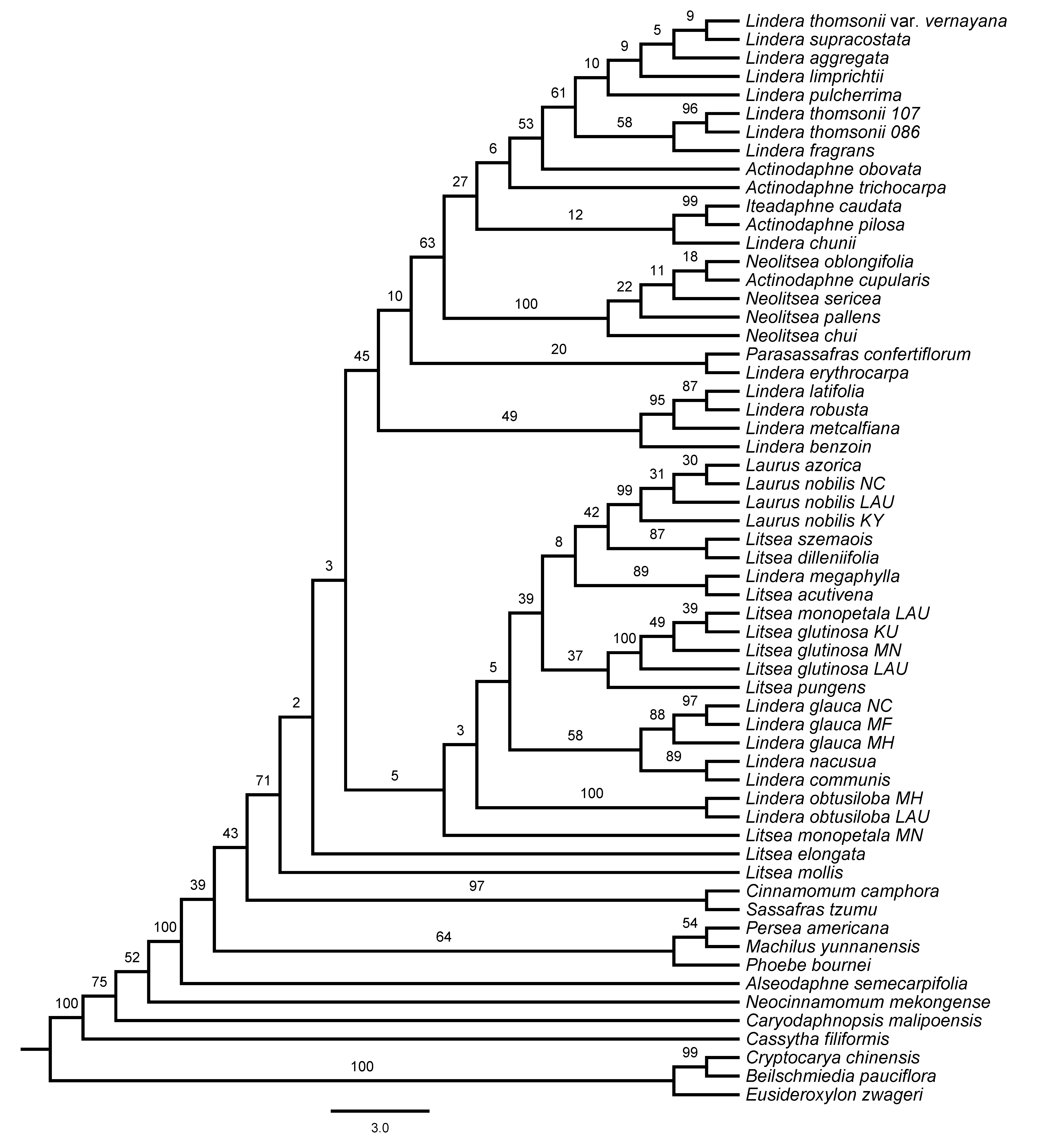

Supplement: Figure S5 — Bootstrap support is indicated on the branches. [file peerj-08-10155-s011.png]

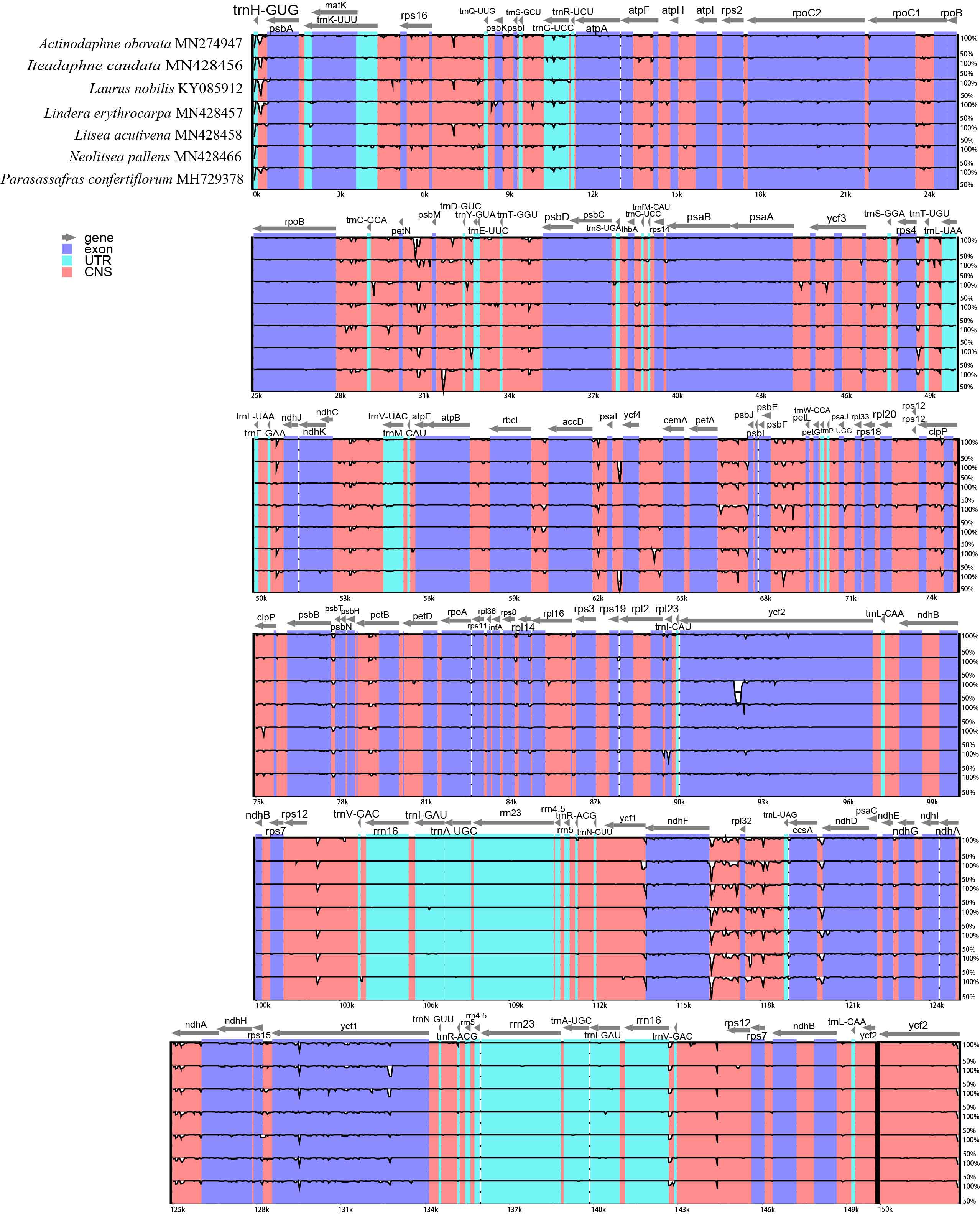

Supplement: Figure S6 — The vertical scale indicates percentage of identity ranging from 50% to 100%. Exons are in dark blue, non-Coding Sequences (CNS) are in red, tRNA and rRNA genes (UTR) are in green. [file peerj-08-10155-s012.jpg]

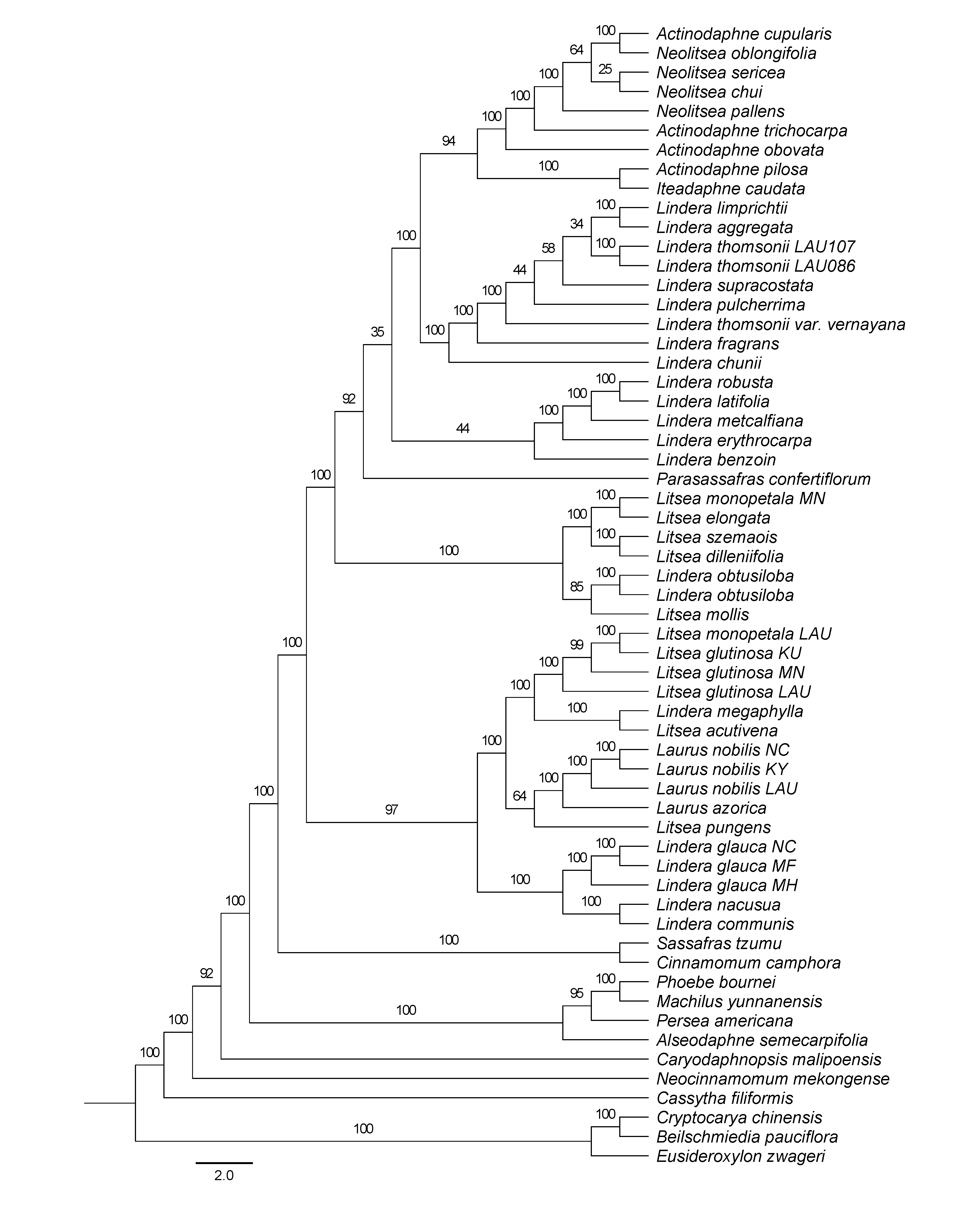

Supplement: Figure S7 — Bootstrap support is indicated on the branches. [file peerj-08-10155-s013.png]

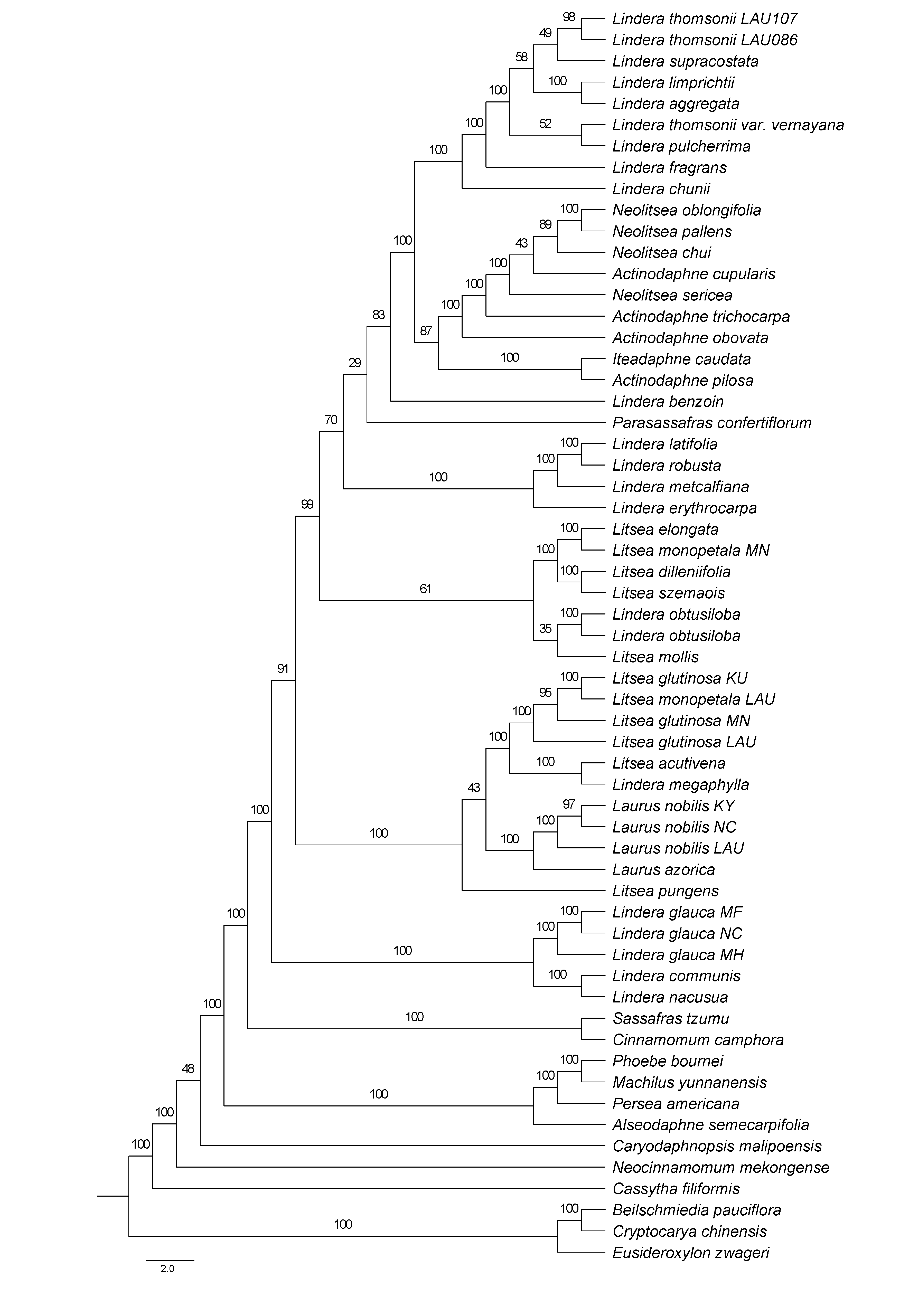

Supplement: Figure S8 — Bootstrap support is indicated on the branches. [file peerj-08-10155-s014.png]

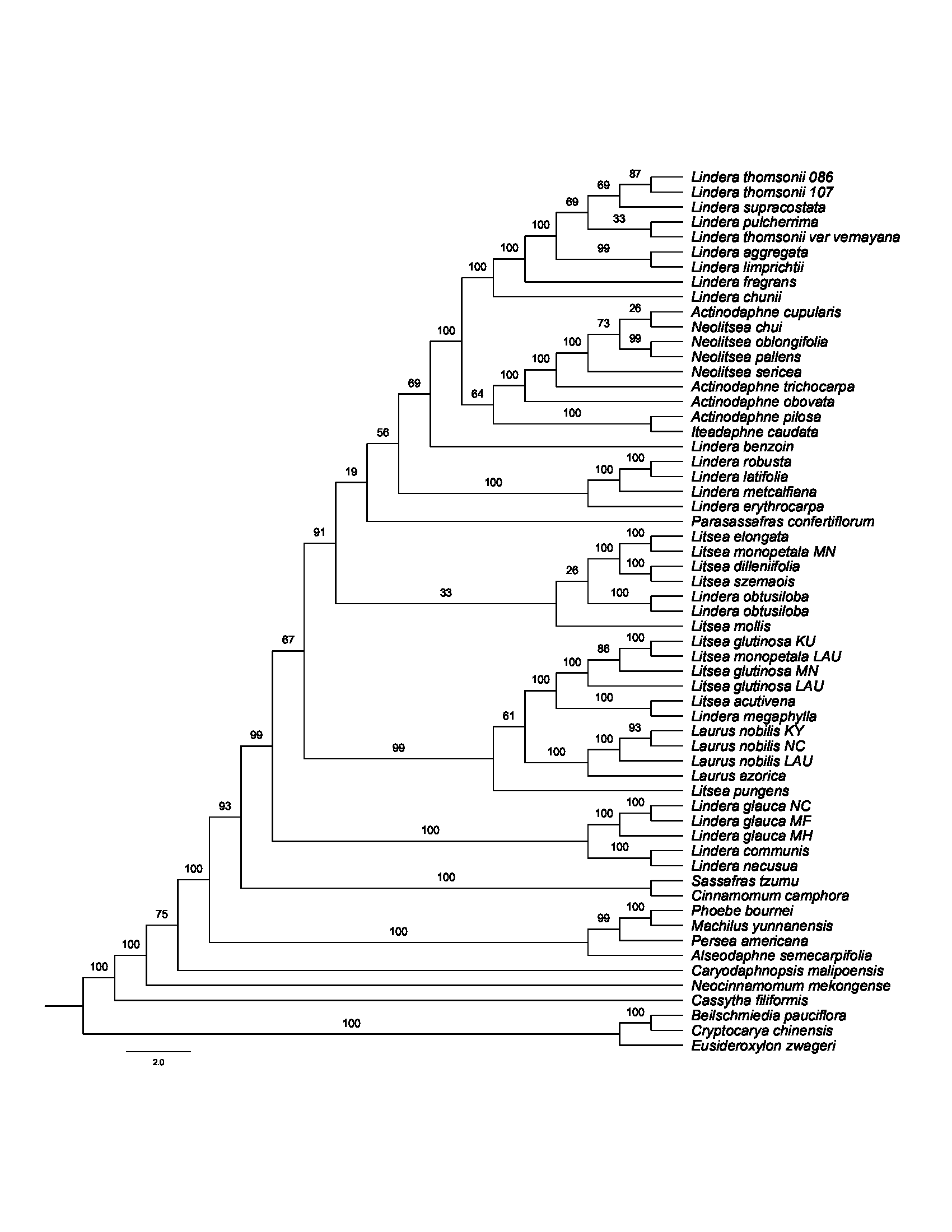

Supplement: Figure S9 — Bootstrap support is indicated on the branches. [file peerj-08-10155-s015.png]

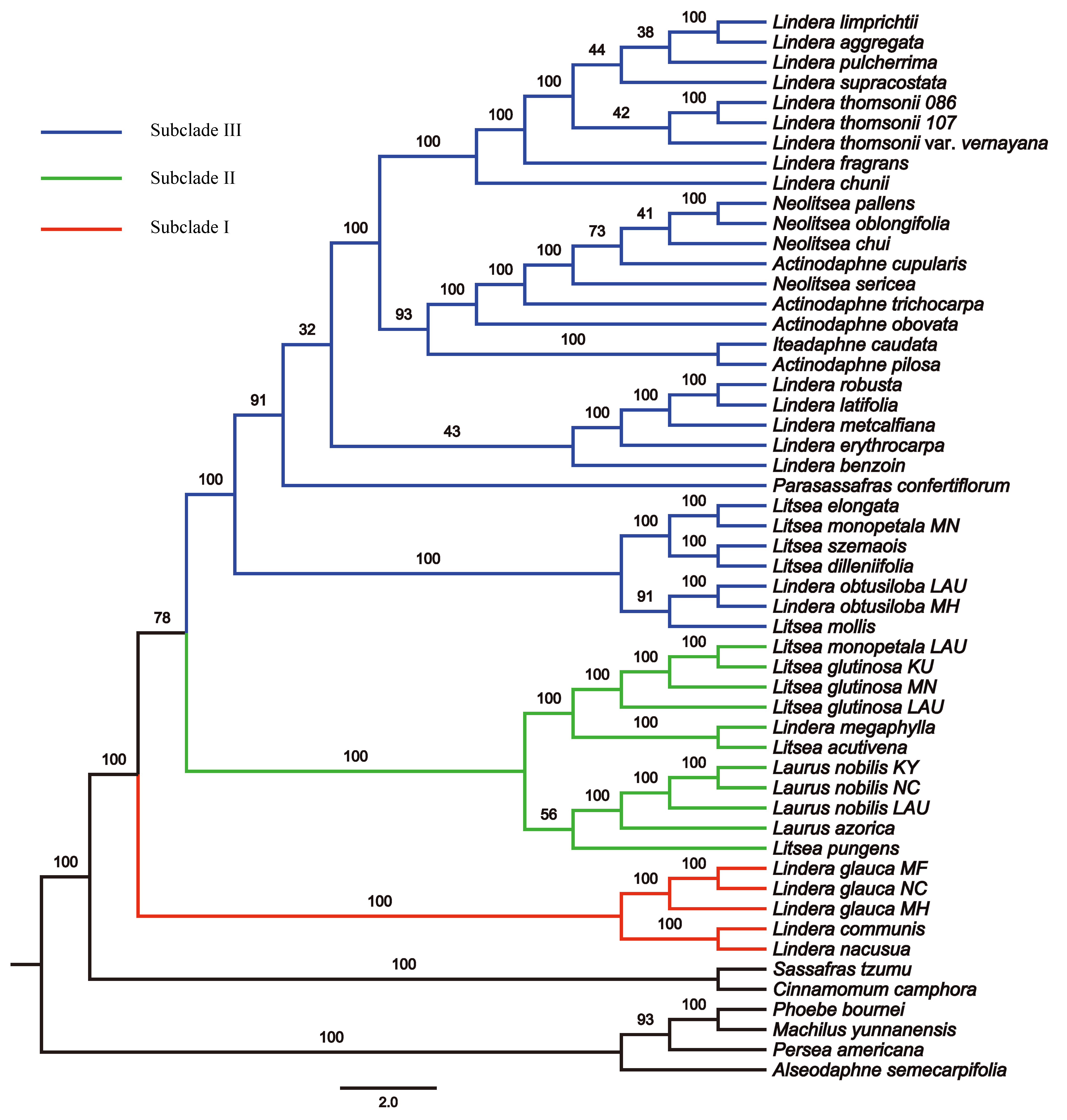

Supplement: Figure S10 — Bootstrap support is indicated on branches. Subclades I, II and III are in red, green and blue, respectively. [file peerj-08-10155-s016.png]

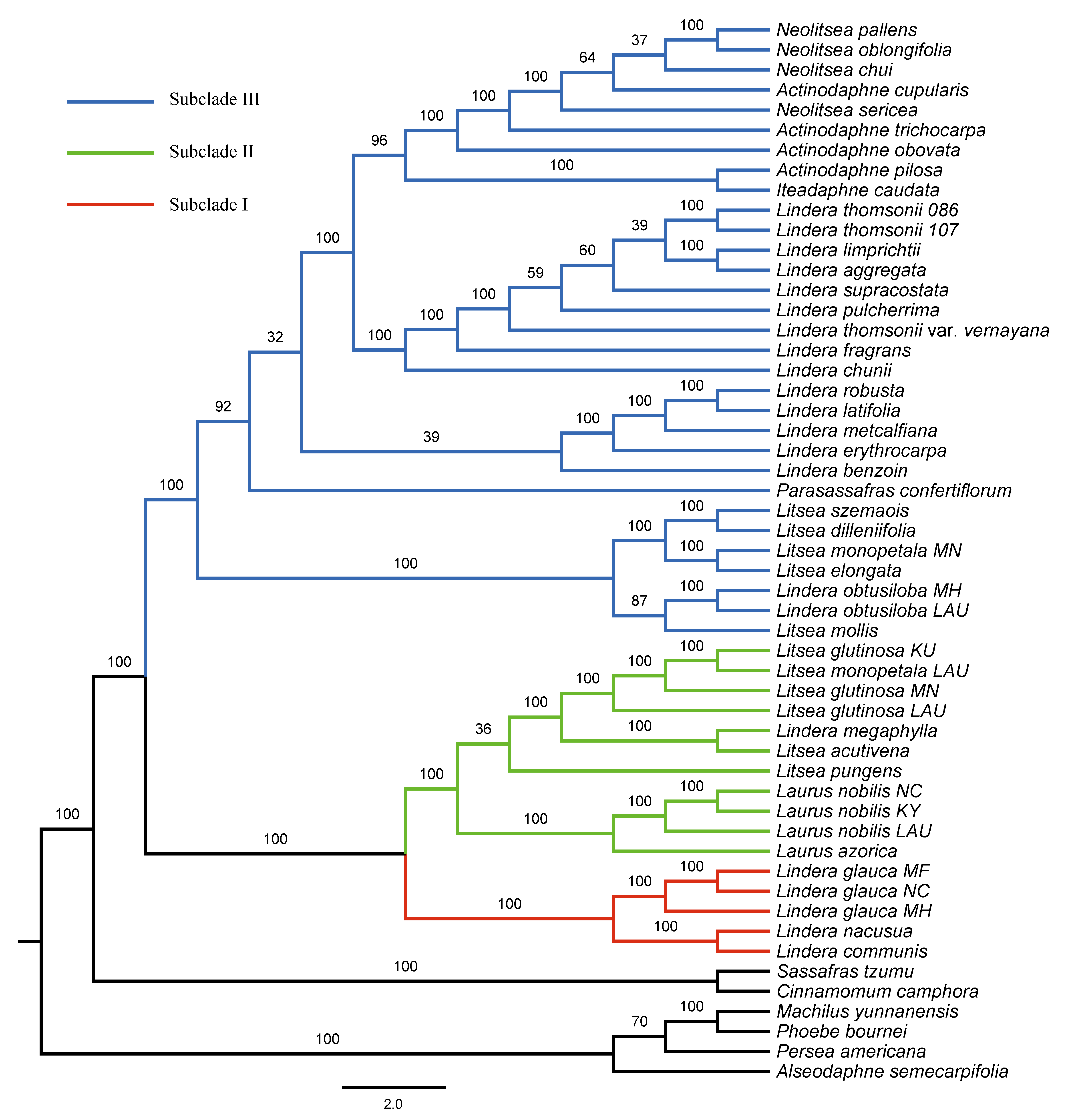

Supplement: Figure S11 — Bootstrap support is indicated on branches. Subclades I, II and III are in red, green and blue, respectively. [file peerj-08-10155-s017.png]
